# Freshwater alga *Raphidocelis subcapitata* undergoes metabolomic changes in response to electrostatic adhesion by micrometer-sized nylon 6 particles

**DOI:** 10.1007/s11356-021-15300-8

**Published:** 2021-07-08

**Authors:** Satomi Mizukami-Murata, Yuji Suzuki, Kensuke Sakurai, Hiromasa Yamashita

**Affiliations:** 1 Water Quality Team, Water Environment Research Group, Public Works Research Institute, Minamihara, 1-6, Tsukuba, Ibaraki, 305-8516 Japan; 2 Innovative Materials and Resources Research Center, Public Works Research Institute, Minamihara, 1-6, Tsukuba, Ibaraki, 305-8516 Japan

**Keywords:** Powdered nylon 6, *Raphidocelis subcapitata*, Growth inhibition, Electrostatic adhesion, Metabolomic analysis, Amino acid catabolic pathway, γ-glutamyl cycle

## Abstract

Nylon powders are a type of microplastic (MP) used in personal care products such as cosmetics and sunscreens. To determine the effects of nylon polymers on freshwater microalgae, we investigated the effects of two types of micrometer-sized nylon polymers, i.e., powdered nylon 6 (Ny6-P) and nylon 12 (Ny12), and four other micrometer-sized MPs, i.e., low-density polyethylene, polyethylene terephthalate, polystyrene, and ultra-high-molecular-weight polyethylene, on the microalga *Raphidocelis subcapitata*. The results showed that Ny6-P inhibited *R. subcapitata* growth more than the other MPs; *R. subcapitata* growth was inhibited by 54.2% with 6.25 mg/L Ny6-P compared with the control. Ny6-P in the culture media adhered to *R. subcapitata* cells electrostatically, which may have disrupted growth and photosynthetic activity. Metabolomic analysis revealed that many metabolites related to the amino acid catabolic pathway and γ-glutamyl cycle were induced, which might trigger responses to avoid starvation and oxidative stress. Our study provides important information on the effects of Ny6-P on algae in freshwater environments.

**Supplementary Information:**

The online version contains supplementary material available at 10.1007/s11356-021-15300-8.

## Introduction

Since the 1950s, the production and use of plastics has increased globally, which has affected the environment, especially in terms of increased amounts of microplastics (MPs) released into aquatic environments (Monteleone et al. 2019). MPs are defined as plastic particles sized <5 mm and can be further categorized based on the production process. Primary MPs are produced as plastic resin pellets or granules that are often added to personal care products, which may flow into aquatic environments mainly via domestic wastewater (Thompson et al. 2004; Mintenig et al. 2017). Secondary MPs are generated as a result of the degradation of larger plastic products over time due to physical, biological, and chemical weathering processes (Li et al. 2016). The use of plastics has resulted in MP contamination in aquatic environments and is drawing attention worldwide. In addition to their presence in marine environments, MPs have been detected at concentrations ranging from 0.00297 to 2.58 g/L in freshwater environments, including rivers, lakes, and wastewater treatment plants, in North America, Asia, Europe, and Australia (Eerkes-Medrano et al. 2015; Rezania et al. 2018; Li et al. 2019). Moreover, various types of MP polymers, such as polyamide (PA), polyethylene (PE), PE terephthalate (PET), polypropylene (PP), and polystyrene (PS), have been detected in these regions (Li et al. 2019; Rezania et al. 2018).

Understanding the effects of MPs on microalgae is essential considering microalgae comprise the base of the food chain in aquatic environments. Many studies have investigated the effects of MPs on freshwater and marine algae in recent years (Bhattacharya et al. 2010; Besseling et al. 2014; Davarpanah and Guilhermino 2015; Sjollema et al. 2016; Bergami et al. 2017; Zhang et al. 2017; Canniff and Hoang 2018; Chae et al. 2018; Mao et al. 2018; Prata et al. 2019; Yi et al. 2019). Most previous research has focused on the effects of PS on algal cells (Bhattacharya et al. 2010; Casado et al. 2013; Besseling et al. 2014; Sjollema et al. 2016; Bergami et al. 2017; Chae et al. 2018; Yi et al. 2019). Nanometer-sized PS particles have been reported to inhibit algal photosynthesis and growth, whereas micrometer-sized PS particles did not pose such effects (Besseling et al. 2014; Sjollema et al. 2016; Yi et al. 2019). Moreover, aggregation of positively charged PS particles was observed when algal cells coexisted, which induced structural damage and oxidative stress in algae; these effects were greater than those of negatively charged PS particles (Bhattacharya et al. 2010; Bergami et al. 2017). The effects of different plastic types other than PS on algal cells have also been reported. Micrometer-sized polyvinyl chloride (PVC) was shown to inhibit microalgal growth via heteroaggregation, which resulted in physical damage to the cells (Zhang et al. 2017; Song et al. 2020). Relatively large-sized PE particles (diameter, 63–75 μm) were reported to enhance algal growth, whereas small-sized PE particles (1–5 μm) had no effects on algae (Davarpanah and Guilhermino 2015; Canniff and Hoang 2018). However, comparison of the toxic effects of different plastic types is difficult since the experimental conditions and tested algae species varied among studies. Additionally, previous reports focused on nanometer-sized particles, which are smaller than microalgae. Micrometer-sized MPs, which are larger than algal cells, are commonly found in aquatic environments; thus, the effects of micrometer-sized MPs on algal cells should be investigated (Eerkes-Medrano et al. 2015; Rezania et al. 2018; Li et al. 2019).

Polyamide (nylon polymers) particles are used in personal care products such as face powder and eyeshadow as opacifying and skin-improving agents (Timm et al. 2011; Burnett et al. 2014). Thus, nylon MPs can be introduced into freshwater environments through human activities such as swimming as well as the influx of domestic wastewater. Nylon polymers are common MPs that are detected in aquatic environments, and their impact on algal cells cannot be ignored (Coyle et al. 2020; Imhof et al. 2016; Mintenig et al. 2017; Li et al. 2019; Scopetani et al. 2019; Yan et al. 2019). Micrometer-sized PA particles have been detected in sewage effluents from wastewater treatment plants and in freshwater fish (Mintenig et al. 2017; Wagner et al. 2019). However, the effects of nylon polymers on algal cells have not yet been elucidated.

The aim of this study was to determine the effect of nylon polymers on the freshwater microalga *Raphidocelis subcapitata*. *R. subcapitata* is widely distributed in freshwater environments and serves as a typical model phytoplankton species for toxicology testing (OECD guidelines 2011). To compare the effects of nylon polymers and other MPs on algal cells, we examined the effects of seven types of MPs—nylon 6 (Ny6), nylon 12 (Ny12), low-density PE (LDPE), PET, PP, PS, and ultra-high-molecular-weight-PE (UHPE)—on *R. subcapitata* growth and photosynthetic activity. Many everyday items are produced from these materials (Li et al. 2016); styrene foams used for food packaging are composed of PS, shopping bags are composed of LDPE, items such as skis and climbing ropes are composed of UHPE, and bottles and lids are composed of PET and PP, respectively. Ny6 and Ny12 are used extensively to produce textile fibers in addition to personal care products. In this study, we evaluated powdered MPs [LDPE, powdered Ny6 (Ny6-P), Ny12, PET, PS, and UHPE] and granule-type MPs [granule Ny6 (Ny6-G) and PP]. The adhesion of *R. subcapitata* by Ny6-P was evaluated by microscopic observation and by measuring electronic potentials. In addition, we employed metabolomic analysis, which is one of the powerful omics tools (i.e., genomics, proteomics, and metabolomics) used to elucidate organism response mechanisms under stress conditions. Metabolomic analysis is used to comprehensively understand metabolic networks in organisms that are affected by environmental factors such as nutrition and chemicals (Carrera et al. 2021; Clish 2015). Many studies have examined the effects of MPs on organisms such as fish, shellfish, and plants, but few have employed metabolomics in algal studies (Qiao et al. 2019; Ding et al. 2020; Wu et al. 2020; Teng et al. 2021). Here, we performed metabolomic analysis to expand our understanding of the biochemical mechanisms of *R. subcapitata* responses to Ny6-P adhesion.

## Materials and methods

### MPs

Six powdered MPs (LDPE, Ny6-P, Ny12, PET, PS, and UHPE) and two granule-type MPs (Ny6-G and PP) were purchased from Goodfellow Cambridge Ltd. (Japan) for this experiment. The maximum particle size of Ny6-P and Ny12 was 50 μm (average diameters: Ny6, 15–20 μm; Ny12, 25–30 μm). Four MPs (LDPE, PET, PS, and UHPE; diameter < 300 μm) were used after fractionation with a 53-μm stainless-steel mesh sieve. Two granule-type MPs were used, Ny6-G and PP (average diameter: 3 mm). All MPs were white in color and contained no additives.

### Test species and culture conditions

The green alga *R. subcapitata* (NIES-35) was obtained from the Microbial Culture Collection of the National Institute for Environmental Studies (NIES) of Japan. The size of *R. subcapitata* cells was 5–12 μm. *R. subcapitata* was cultured in AAP medium sterilized by membrane filtration (0.22-μm pore size) in a sterilized flask (OECD guidelines 2011). Algal cells were cultured at 25 ± 1 °C on a rotating shaking device at 100 rpm (Taitec Co., NR-80, Japan) in an incubator under white fluorescent light [3000 Lux, measured using an illuminance meter (mobiken Lx2, Sanwa Co., Japan)] with a 16-h/8-h light/dark cycle. Algal cells in AAP medium were subcultured every week. Additionally, *R. subcapitata* was cultured in C medium under the same culture conditions to yield a higher concentration of cells for the algal adhesion tests and metabolomic analysis (NIES collection 2001). Algal cells in C medium were subcultured every 2 weeks.

### Algal growth and photosynthesis inhibition test

Four Ny6-P concentrations (6.25, 12.5, 25, and 50 mg/L) and three Ny12 concentrations (150, 350, and 750 mg/L) were tested. Four powdered MPs (LDPE, PET, PS, and UHPE) were examined at a concentration of 750 mg/L. The granule-type MPs were tested at a concentration of 7500 mg/L (13 Ny6-G particles, 6 PP particles) because the particle weights of Ny6-G and PP were approximately 11.5 mg and 24.5 mg, respectively. *R. subcapitata* cells were incubated for 72 h until reaching log-phase growth and then added to a flask containing AAP medium at an initial cell density of 1 × 10^4^ cells/mL. The samples were cultured in an incubator for 72 h at 25 ± 1 °C under constant illumination at 4000 Lux on a rotating shaking device at 100 rpm. The flasks were positioned randomly for incubation. Samples with only algal cells (without MPs) were used as the control. In the Ny6-P and Ny12 treatments, algal cell numbers were determined every 24 h using a cell counter (CDA-1000B, Sysmex Co., Japan). In the LDPE, PET, PS, UHPE, Ny6-G, and PP treatments, algal cell numbers were determined after 72 h. For the analysis of photosynthetic activity, the chlorophyll-a (Chl-a) contents of each flask were measured after 72 h of exposure. All experiments were performed in triplicate.

### Measurement of Chl-a content

After exposure of *R. subcapitata* to MPs for 72 h, the culture solutions were filtered (GF/C, Whatman) and the filter papers were stored at −30 °C until further analyses. Filtered samples were ground using a mortar with 10 mL of acetone (90% concentration with Milli-Q water, Fujifilm Wako Pure Chemical Co., Ltd., Japan) and stored at 4 °C overnight to extract Chl-a. Supernatants were obtained by centrifuging twice at 1500×*g* for 10 min. The Chl-a content was determined using a ultraviolet–visible recording spectrophotometer (UV-160, Shimadzu Inc., Japan) based on the absorption technique described by Lorenzen (1967). The absorbances of the extracted samples were measured at 665 nm and 750 nm to determine the Chl-a content.

### Nylon polymer adhesion tests

**Fig. 1:**
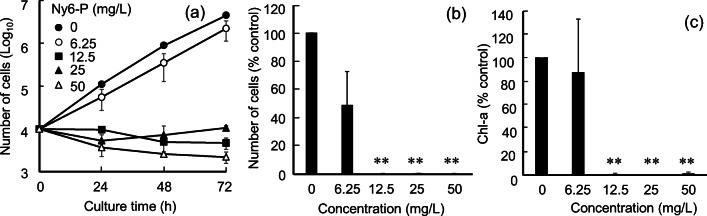
Effects of powdered nylon 6 (Ny6-P) on *Raphidocelis subcapitata*. **a** Growth dynamics of *R. subcapitata* exposed to Ny6-P at concentrations of 6.25, 12.5, 25, and 50 mg/L for 72 h*.* **b** Cell numbers and **c** chlorophyll-a (Chl-a) contents after the same exposure times. Data are shown as mean ± standard deviation. A single asterisk (*) and double asterisks (**) represent significant differences relative to the controls at *P* < 0.05 and *P* < 0.01, respectively

The Ny6-P EC_50_ for *R. subcapitata* cells (1 × 10^4^ cells/mL) was calculated as 5–6 mg/L using data shown in Fig. 1a (6.25 mg/L Ny6-P reduced *R. subcapitata* growth by 54.2%). Based on these results, algal cell and nylon polymer concentrations approximately 100-fold higher were used in the adhesion experiment to enable naked-eye observations. Ny6-P or Ny12 (500 mg/L) was added to the C medium, and algal cell culture solution was added to each flask at an initial cell density of 1 × 10^6^ cells/mL. Each sample was incubated at 25 ± 1 °C under constant illumination (4000 Lux) on a rotating shaking device at 100 rpm. Algal cells without MPs were used as the control. The number of particles in the supernatant (A), including both algal cells and nylon particles, was measured at five time points (0, 30, 90, 240, and 300 min) using a cell counter. A medium with only nylon particles was also prepared, and the number of these particles (B) was measured at each of the five time points. The number of algal cells in the supernatant was calculated by subtracting (B) from (A). After incubation for 300 min, precipitates in each flask were observed using an optical microscope (BX51, Olympus Co., Japan). All experiments were performed in triplicate.

### Zeta potential measurement

*R. subcapitata* cells (8 × 10^4^ cells/mL) and 5 mg/L Ny6-P in AAP medium were stirred for 1 min to allow Ny6-P adhesion to *R. subcapitata* cells. After stirring, the samples were allowed to stand for 1 min, and the zeta potential was then measured. Approximately 1 mL of each sample was injected into the cuvette for zeta potential analysis, which was conducted at 20 °C using a Zeta Potential and Submicron Particle Size Analyzer (Delsa™Nano HC, Beckman Coulter Inc., Japan). All experiments were performed in triplicate.

### Metabolomic analysis

*R. subcapitata* cells (1 × 10^4^ cells/mL) were treated with Ny6-P (6 mg/L) in C medium and incubated at 25 ± 1 °C under constant illumination (4000 Lux) on a rotating shaking device at 100 rpm. After 0, 6, and 24 h of treatment, algal cells (3 × 10^7^ cells) were collected by filtration using 1.0-μm pore-sized Omnipore™ membrane filters (hydrophilic PTFE, Merck Millipore, UK) and washed twice with Milli-Q water. The filters were then soaked in 2.0 mL of methanol containing Milli-Q water and internal standards (H3304-1002, Human Metabolome Technologies [HMT], Japan) and ultrasonicated for 30 s. Cell suspensions were stored at −80 °C until further analysis. The extract was obtained with cell disruption and centrifuged at 2300×*g* at 4 °C for 5 min. Then, 700 μL of the upper aqueous layer was centrifugally filtered through a Millipore 5-kDa cutoff filter at 9100×*g* at 4 °C for 120 min to remove proteins. The filtrate was concentrated by centrifugation and resuspended in 50 μL of Milli-Q water for capillary electrophoresis time-of-flight mass spectrometry (CE-TOFMS) analysis.

Metabolome analysis was performed using CE-TOFMS (Ohashi et al. 2008; Ooga et al. 2011). Briefly, CE-TOFMS analysis was conducted using an Agilent capillary electrophoresis system equipped with an Agilent 6210 time-of-flight mass spectrometer (Agilent Technologies, Germany). The systems were controlled using Agilent G2201AA ChemStation software version B.03.01 for CE (Agilent Technologies) and connected by a fused silica capillary tube (50 μm i.d. × 80 cm total length) with commercial electrophoresis buffers (H3301-1001 and I3302-1023 for cation and anion analyses, respectively, HMT) as the electrolyte. The spectrometer was scanned from m/z 50 to 1000. Peaks were extracted using MasterHands automatic integration software (Keio University, Japan; Sugimoto et al. 2009) and MassHunter Quantitative Analysis B.04.00 (Agilent Technologies) to obtain m/z, peak area, and migration time (MT). Signal peaks were annotated according to the metabolite database based on their m/z values and MTs. Annotated peak areas were then normalized based on the internal standard and sample amounts to obtain relative levels of each metabolite. Principal component analysis was performed using PeakStat and SampleStat, which are HMT’s proprietary software. Algal cells without MPs were used as the control. The time-course experiment was performed once.

### Statistical analysis

Data were expressed as the mean ± standard deviation of three independent experiments. Statistical differences between control and treated algal cells were determined using *t*-tests. Significance levels were set at *P* < 0.05 and *P* < 0.01.

## Results and discussion

### Effects of nylon polymers on *R. subcapitata*

The effects of nylon polymers on *R. subcapitata* were evaluated using two types of nylon polymers, Ny6-P and Ny12. Ny6-P and Ny12 are linear polymers with amide fusion, although some of their chemical characteristics differ: Ny6-P has higher water absorption and heat resistance than Ny12 [polyamide-nylon6 (PA6) material information 2021; polyamide-nylon12 (PA12) material information 2021]. Figure 1a, b shows *R. subcapitata* growth under various Ny6-P concentrations. Algal growth was inhibited with increasing Ny6-P concentration (Fig. 1a). At 72 h, the number of algal cells observed under the condition with 6.25 mg/L of Ny6-P was 54% lower than that observed under the control condition, although the difference was not statistically significant (*P* = 0.06, Fig. 1b). Interestingly, the number of algal cells decreased under the 12.5, 25, and 50 mg/L Ny6-P treatments; after 72 h, the number of cells was reduced by 88.3%, 76.7%, and 95.0%, respectively, compared with the 0-h control (1 × 10^4^ cells/mL). The Chl-a contents of *R. subcapitata* treated with Ny6-P were also examined (Fig. 1c). The Chl-a contents decreased with increasing Ny6-P concentration, following a similar trend as algal cell growth; a small amount of Chl-a was detected in *R. subcapitata* cells treated with Ny6-P concentrations >12.5 mg/L (inhibition rate: 95.4%; *P <* 0.01). These results demonstrate that Ny6-P has the capacity to inhibit *R. subcapitata* cell growth.

**Fig. 2:**
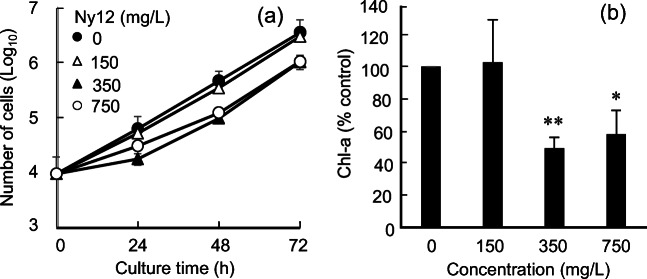
Effects of nylon 12 (Ny12) on *Raphidocelis subcapitata*. **a** Growth dynamics and **b** chlorophyll-a (Chl-a) contents after exposure of *R. subcapitata* to Ny12 at concentrations of 150, 350, and 750 mg/L for 72 h*.* Data are shown as mean ± standard deviation. A single asterisk (*) and double asterisks (**) represent significant differences relative to the controls at *P* < 0.05 and *P* < 0.01, respectively

Figure 2 shows the growth of *R. subcapitata* cells treated with Ny12. Algal cell growth was inhibited with increasing Ny12 concentration, but a high concentration of Ny12 (more than 350 mg/L) was required to inhibit cell growth compared with 6.25 mg/L Ny6-P (Fig. 2a). The number of algal cells was reduced by 20.4%, 70.9%, and 63.8% after 72 h of treatment with 150, 350, and 750 mg/L Ny12, respectively, compared with the control. Similarly, the Chl-a content decreased with increasing Ny12 concentration (Fig. 2b). After 72 h, the *R. subcapitata* Chl-a content in the 350 mg/L Ny12 treatment was reduced by 49.2% compared with that in the control (*P <* 0.01). These results show that Ny6-P has a greater capacity to inhibit *R. subcapitata* growth than Ny12. Moreover, Ny6-P particles sank to the bottom of the flasks more easily than Ny12 particles (data not shown). Ny6-P was uniformly dispersed in the medium, whereas Ny12 formed uneven aggregates after 72 h of treatment. These differences in dispersion may reflect differences in inhibitory effects on *R. subcapitata* between Ny6-P and Ny12.

The effects of granule-type MPs (Ny6-G and PP) on *R. subcapitata* were investigated (Supplementary data Fig. S1). Under the experimental conditions, no significant decreases in growth and photosynthetic activity (Chl-a content) were observed in the Ny6-G or PP treatment, even at the highest concentration (7500 mg/L). These results suggest that millimeter-sized MPs do not inhibit *R. subcapitata* growth. Block-type PVC (1 mm) did not affect *Skeletonema costatum* growth compared with powdered PVC (1 μm) (Zhang et al. 2017). Micrometer-sized PS (5–6 μm) did not affect *Chlorella pyrenoidosa* growth compared with nanometer-sized PS (0.55 μm) (Sjollema et al. 2016; Yi et al. 2019). Our results also indicate that the particle size of Ny6 (powder: average diameter = 15–20 μm; granule: average diameter = 3 mm) is an important indicator of the potential effects on algal growth.

### Effects of four types of MPs on *R. subcapitata*

**Fig. 3:**
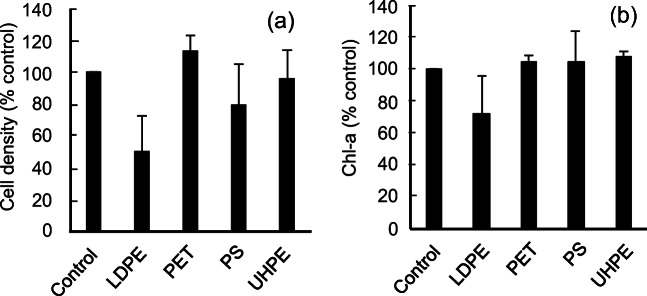
Effects of four powdered microplastics (MPs) on *Raphidocelis subcapitata*. Comparison of **a** cell numbers and **b** chlorophyll-a (Chl-a) contents of *R. subcapitata* exposed to low-density polyethylene (LDPE), PE terephthalate (PET), polystyrene (PS), and ultra-high-molecular-weight PE (UHPE) at 750 mg/L for 72 h*.* Error bars represent standard deviations

To compare the effects of nylon polymers and other MPs on *R. subcapitata*, we examined the effects of powdered LDPE, PET, PS, and UHPE on *R. subcapitata*. Figure 3a, b show the ratios of cell densities and Chl-a contents of *R. subcapitata* treated with each MP compared with the corresponding control conditions. PET, PS, and UHPE did not significantly affect *R. subcapitata* growth or Chl-a content, even at the highest concentration of 750 mg/L. After 72 h, only LDPE decreased the number of algal cells and Chl-a content by 50.4% and 27.9%, respectively, although the differences were not statistically significant. Our results indicate that these MPs had limited effects on *R. subcapitata* growth and photosynthetic activity. Among all experimental conditions, Ny6-P had the greatest inhibitory effect on *R. subcapitata* growth, followed by Ny12, LDPE, PS, UHPE, and PET.

Previous studies have reported that micrometer-sized MPs affect algal growth (Table 1). For example, PVC had higher capacity to inhibit algal growth; 50 mg/L PVC (1 μm) inhibited the growth of *S. costatum* by 39.7% and 200 mg/L PVC (74 μm) inhibited the growth of *Phaeodactylum tricornutum* MASCC-0025 by 21.2% after 96 h of exposure (Zhang et al. 2017; Zhu et al. 2019; Song et al. 2020). Moreover, 1-μm PS (100 mg/L) inhibited the growth of *C. pyrenoidosa* by 38.1% after 22 days of exposure (Mao et al. 2018). In contrast, PE promoted the growth of *Chlorella* sp. and *R. subcapitata* (Canniff and Hoang 2018; Song et al. 2020). Compared with the previous reports, our results suggest that micrometer-sized Ny6-P is one of the MPs which has a high capacity to inhibit algal growth, although no direct comparisons could be made as no other study used the same material tested here.

**Table 1:** Effects of micrometer-sized microplastics on algae

Microplastic*	Size (μm)	Maximum concentration (mg/L)	Environment	Alga	Exposure time	Effects	Reference
PS	6	250	Salt water	*Dunaliella tertiolecta*	72 h	No effect	Sjollema et al. 2016
PS	5	60	Freshwater	*Chlorella pyrenoidosa*	96 h	No effect	Yi et al. 2019
PS	1	100	Freshwater	*Chlorella pyrenoidosa*	22 days	Growth inhibition	Mao et al. 2018
PVC	1	50	Salt water	*Skeletonema costatum*	96 h	Growth inhibition	Zhang et al. 2017
PVC	1000	2000	Salt water	*Skeletonema costatum*	96 h	No effect	
PE, PET, and PVC	74	200	Freshwater	*Chlorella sp. L38*	96 h	Growth promotion	Song et al. 2020
PE, PET, PP, and PVC	74	200	Salt water	*Phaeodactylum tricornutum MASCC-0025*	96 h	Growth inhibition	
PE, PS, and PVC	74	100	Salt water	*Skeletonema costatum*	96 h	Growth inhibition	Zhu et al. 2019
PE	130	36–75	Freshwater	*Raphidocelis subcapitata*	5 days	Growth promotion	Canniff and Hong 2018
PP	400–1000	400	Freshwater	*Chlamydomonas reinhardtii*	78 days	Growth inhibition	Lagarde et al. 2016
HDPE	400–1000	400	Freshwater	*Chlamydomonas reinhardtii*	78 days	No effect	

^*^High-density PE, HDPE; polyethylene, PE; PE terephthalate, PET; polypropylene, PP; polystyrene, PS; polyvinyl chloride, PVC

### Adhesion of *R. subcapitata* cells and nylon particles

**Fig. 4:**
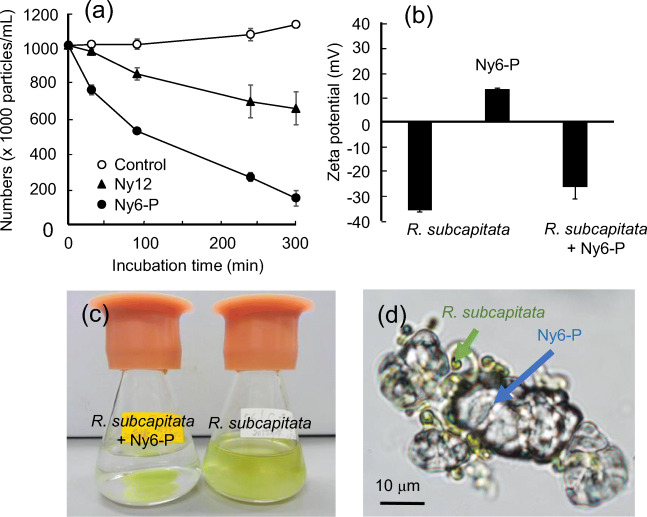
Nylon particle adhesion to *Raphidocelis subcapitata* cells. **a** Dynamics of algal cells treated with powdered nylon 6 (Ny6-P) or nylon 12 (Ny12) in culture supernatants. **b** Zeta potential of *R. subcapitata*, Ny6-P, and *R. subcapitata* mixed with Ny6-P (*R. subcapitata* + Ny6-P). **c** Culture media containing *R. subcapitata* in flasks after 300 min of stirring with or without Ny6-P treatment. **d** Micrograph of Ny6-P adhering to *R. subcapitata* cells

Nylon polymers had more inhibitory effects on *R. subcapitata* growth than the four other types of MPs. To gain deeper understanding of the phenomena underlying this observation, we performed further experiments using Ny6-P and Ny12. Figure 4a shows the number of algal cells in the supernatant of media treated with each nylon polymer. The number of floating algal cells in the supernatant immediately decreased with Ny6-P treatment, and the number of cells was reduced by 87% compared with the control after 5 h of incubation. In this treatment, particles of green-colored Ny6-P, which adhered to many algal cells, were observed at the bottom of the flask, whereas the supernatant was transparent (Fig. 4c, d). In the Ny12 treatment, the number of floating algal cells in the supernatant gradually decreased with time; the number of cells was reduced by 42.3% after 5 h of incubation (Fig. 4a). Ny12 precipitates were also observed at the bottom of flasks with a slight green color (data not shown). Comparative analysis of the number of algal cells in the supernatant treated with each nylon polymer suggested that Ny6-P has a higher capacity to cause adhesive interaction with *R. subcapitata* cells than Ny12. Under the experimental conditions, one Ny6-P particle was estimated to attract 7.4 algal cells to adhere after 300 min of incubation.

Nanometer-sized MPs, including PS and PVC, have been reported to adsorb to algal cell surfaces (Zhang et al. 2017; Mao et al. 2018; Yi et al. 2019). Our results show that micrometer-sized Ny6-P particles possess the ability to adhere *R. subcapitata* cells. Figure 4b shows the zeta potentials of Ny6-P and *R. subcapitata* cells, which were measured to quantify the adhesion characteristics of Ny6-P to *R. subcapitata* cells. Ny6-P and *R. subcapitata* cells in media had zeta potentials of 13.0 mV and − 36.0 mV, respectively. After interaction with Ny6-P, *R. subcapitata* cells (*R. subcapitata* + Ny6-P) exhibited an increased zeta potential (−26.5 mV). PA (nylon 6, 6) is known to be positively charged, whereas typical plastic materials such as PE and PS tend to be negatively charged in triboelectric series (Liu et al. 2015; Kim et al. 2017). Algal cells are also known to be negatively charged (Ewerts et al. 2017). It has been demonstrated that positively charged PS particles (20–50 nm) have a higher binding affinity toward algal cells, which produces a greater effect on the cells than negatively charged PS particles (Bergami et al. 2017; Nolte et al. 2017; Bhattacharya et al. 2010). Based on these results, the present study suggests that positively charged Ny6-P has a binding affinity toward negatively charged *R. subcapitata* and that electrostatic adhesive interaction between them may be one of the causes to inhibit algal cell growth. These findings are consistent with the observed decrease in the number of *R. subcapitata* cells treated with Ny6-P (Fig. 1a).

### Global metabolomic analysis of *R. subcapitata* treated with Ny6-P

**Fig. 5:**
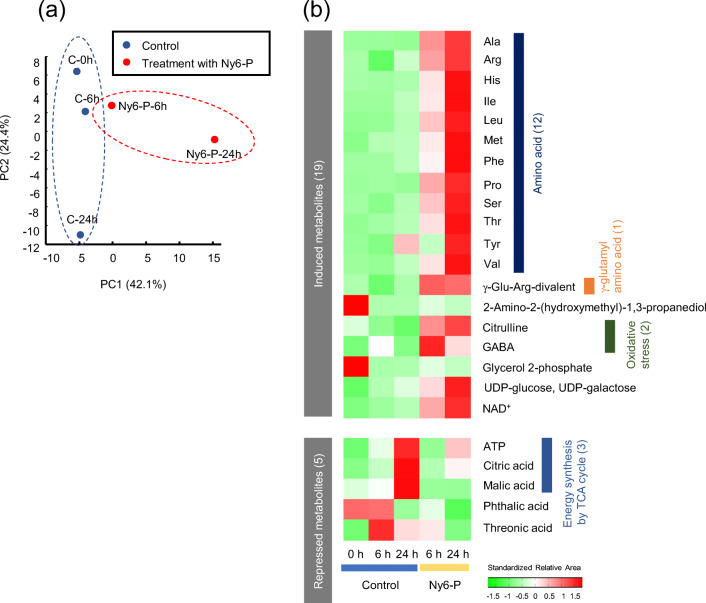
Metabolomic alterations in *Raphidocelis subcapitata* after exposure to Ny6-P. **a** Principal component analysis (PCA) plots of metabolite profiles from *R. subcapitata* treated with Ny6-P. The percentages listed on the axis labels indicate the fraction of variance explained by the first (PC1) and second (PC2) principal components. Ny6-P-6 h and Ny6-P-24 h show plots of *R. subcapitata* treated with Ny6-P for 6 and 24 h, respectively. C-0 h, C-6 h, and C-24 h show plots of *R. subcapitata* without Ny6-P (control) incubated for 0, 6, and 24 h, respectively. **b** Metabolic alterations of *R. subcapitata* treated with Ny6-P. The heatmap shows induced or repressed metabolites in *P. subcapitata* cells exposed to Ny6-P for 6 and 24 h

To elucidate the biochemical mechanism of *R. subcapitata* response to adhesion by Ny6-P, metabolomics analysis was performed using CE-TOFMS. The analysis showed the presence of 177 compounds as primary metabolites (Table S1), which led to the detection of 89 signals in cation mode and 88 signals in anion mode. Figure 5a shows the principal component analysis (PCA) plot of *R. subcapitata* metabolites with and without Ny6-P treatment. As shown in the plot, algal cells treated with Ny6-P were clearly separated from the control group. The first principal component (PC1) accounted for 42.1% of the variation, showing the variation in metabolites resulting from the effects of Ny6-P on *R. subcapitata*, and the second principal component (PC2) accounted for 24.4% of the variation, showing the variation in metabolites during *R. subcapitata* growth. In particular, metabolites related to five amino acids [phenylalanine (Phe), glycine (Gly), methionine (Met), histidine (His), and isoleucine (Ile)] and three gamma-glutamyl (γ-Glu) amino acids [γ-Glu-asparagine (Asn), γ-Glu-His, and γ-Glu-lysine (Lys)_divalent] exhibited the 10 highest factor loadings in PC1 (Table S2). Figure 5b shows the expressed metabolites (19 induced and 5 repressed) in *R. subcapitata* cells treated with Ny6-P for 6 and 24 h. High accumulation of amino acids was observed as an important adjustment of the organism following treatment with Ny6-P. Twelve amino acids, i.e., alanine (Ala), arginine (Arg), His, Ile, leucine (Leu), Met, Phe, proline (Pro), serine (Ser), threonine (Thr), tyrosine (Tyr), and valine (Val), were detected in 19 metabolites with increased expression. Amino acid contents mostly increased with time; particularly, His, Phe, and Pro showed 8.21-, 6.72-, and 6.74-fold increases after 24-h exposure, respectively.

In general, algae generate energy for growth by photosynthetic carbon assimilation under photoirradiation. In contrast, algae accumulate free amino acids as energy sources via autophagy systems under stressors such as starvation and dark conditions, similar to plants and yeast (Izumi et al. 2013; Hildebrandt et al. 2015; Hirota et al. 2018; Mubeen et al. 2018). MPs have been demonstrated to decrease chlorophyll contents in algal cells (Fig. 1c; Song et al. 2020; Zhan et al. 2017). Decreases in ϕPSII activity were also reported in algae treated with MPs (Zhang et al. 2017; Sjollema et al. 2016). The adhesion of MPs onto the surface of algal cells may shield light and impede nutrient intake, thereby disrupting normal photosynthesis and respiration processes. Our metabolomic results suggested that the energy availability was changing from photosynthetic carbon assimilation to amino acid catabolic pathway in *R. subcapitata* after adhesion of Ny6-P particles. Among the repressed metabolites, three amino acids involved in energy synthesis via the tricarboxylic acid cycle were detected (Fig. 5b). At 24 h, citric acid, malic acid, and adenosine triphosphate (ATP) exhibited 0.4-, 0.2-, and 0.6-fold decreases in concentration, respectively, relative to that observed under the control condition. These results may reflect energy starvation resulting from photosynthesis interference.

**Fig. 6:**
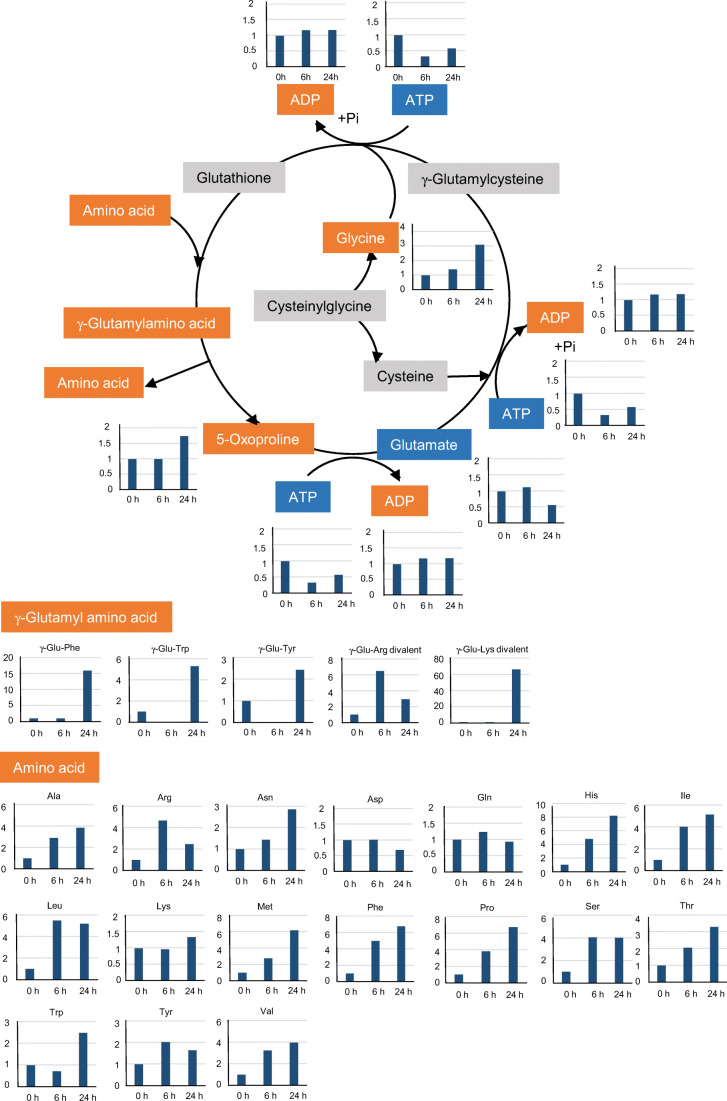
Metabolites related to the γ-glutamyl cycle in *Raphidocelis subcapitata* after exposure to Ny6-P. Orange, induced; blue, repressed; gray, not detected. Vertical axes show fold changes of metabolites in *R. subcapitata* cells treated with Ny6-P compared with the control

As shown in Fig. 6, most metabolites related to the γ-glutamyl cycle were induced with time, although metabolites related to amino acids overlapped in the amino acid catabolic pathway. Four γ-Glu-amino acids, namely γ-Glu-Lys divalent, γ-Glu-Phe, γ-Glu-tryptophan (Trp), and γ-Glu-Tyr, were accumulated at 24 h, while only γ-Glu-Arg-divalent was detected at both 6 and 24 h. These results indicate that the γ-glutamyl cycle may be induced in *R. subcapitata* cells that are adhered on Ny6-P. The γ-glutamyl cycle is considered an antioxidative system that protects against reactive oxygen species (ROS) accumulation in organisms, including plants and bacteria (Masi et al. 2015; Bachhawat and Yadav 2018). This cycle is also responsible for the biosynthesis and utilization of glutathione by amino acid transport systems and uses ATP as energy. MPs have been demonstrated to induce oxidative stress in algae in addition to causing physical damage (Bhattacharya et al. 2010; Mao et al. 2018; Song et al. 2020). Adsorption of nanometer-sized and positively charged PS particles was shown to stimulate ROS production in *Chlorella* and *Scenedesmus* (Bhattacharya et al. 2010). Micrometer-sized PP, PE, PET, and PVC have also produced signs of oxidative stress in *Chlorella* sp. and *P. tricornutum*, as detected via measured malondialdehyde and superoxide dismutase concentrations (Song et al. 2020). Furthermore, PS beads are known to induce electron accumulation from damaged chloroplasts, which causes oxidative stress in *C. pyrenoidosa* (Mao et al. 2018). Some metabolites related to oxidative stress were also detected in this study (Fig. S2). Two such metabolites, citrulline and γ-aminobutyric acid (GABA), were accumulated at 6 and 24 h (Fig. 5b). Citrulline protects DNA and enzymes from oxidative injuries, and GABA restricts ROS accumulation in plants (Akashi et al. 2001; Ruiz et al. 2019). Four other metabolites related to oxidative stress in plants, i.e., cadaverine, dopamine, methionine sulfoxide, and 5-oxoproline, were also detected after treatment for 24 h (Fig. S2) (Aronova et al. 2005; Jacques et al. 2015; Liu et al. 2020; Ohkama-Ohtsu et al. 2008; Shevyakova et al. 2001). Our results suggest that oxidative stress, although not observed directly, was produced in *R. subcapitata* cells adsorbed by Ny6-P, and that the cells responded via the activation of antioxidant systems such as the γ-glutamyl cycle.

**Fig. 7:**
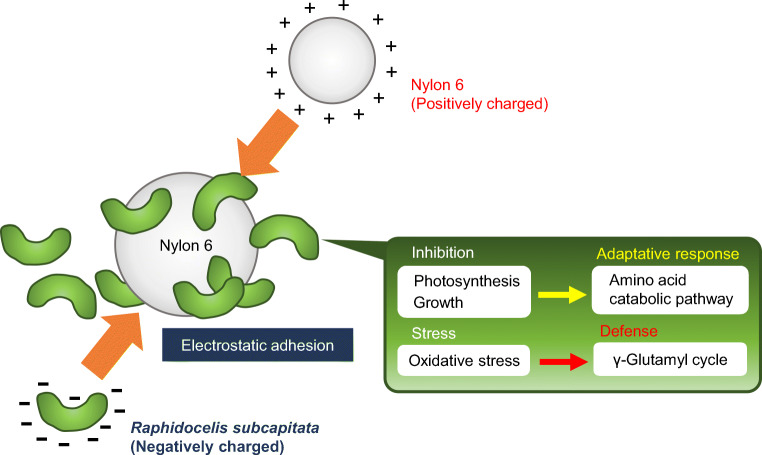
A model diagram of the interaction mechanism between *Raphidocelis subcapitata* and Ny6-P

Figure 7 illustrates the presumed *R. subcapitata* response to treatment with Ny6-P. When Ny6-P was added to the culture solution, *R. subcapitata* electrostatically adhered to the surface of Ny6-P particles. These effects may inhibit *R. subcapitata* photosynthetic activity and growth by shielding light and obstructing nutrient intake. Under this condition, it was indicated that the amino acid catabolic pathway is induced in *R. subcapitata*, which may be an avoidance response to starvation. *R. subcapitata* induced several metabolites related to oxidative stress, including those of the γ-glutamyl cycle, which may be a response by which to reduce stress.

## Conclusion

The effects of micrometer-sized nylon polyamides (Ny6 and Ny12) on *R. subcapitata* were investigated through comparison with other micrometer-sized MPs (i.e., LDPE, PET, PS, and UHPE). Ny6-P had the highest ability to inhibit *R. subcapitata* growth and photosynthetic activity among all tested MPs. The particle size of Ny6 was an important factor: micrometer-sized Ny6-P inhibited *R. subcapitata* growth by 54.2% compared with that of the control at a concentration of 6.25 mg/L, whereas millimeter-sized Ny6-B had no effect on growth even at the highest concentration (7500 mg/L). *R. subcapitata* cells in culture media treated with Ny6-P settled to the bottom of the flask with time, which was caused by electrostatic adhesion: Ny6-P was positively charged whereas *R. subcapitata* cells were negatively charged. Metabolomics analysis was performed to reveal the biochemical mechanism of *R. subcapitata* cells adhered to Ny6-P. PCA plot showed that five amino acids, including Phe, Gly, and three γ-Glu amino acids (including γ-Glu-Asn and γ-Glu-His), exhibited the 10 highest factor loadings in PC1. In the list of expressed metabolites detected in *R. subcapitata* cells treated with Ny6-P for 6 and 24 h, many metabolites included in the amino acid catabolic pathway and γ-glutamyl cycle were induced, which might be related to the responses of starvation and oxidative stress. Our results will help improve understanding of the physiological phenomena that occur in algal cells exposed to Ny6-P in freshwater environments.

## Supplementary Information

ESM 1 (PPTX 46 kb)

ESM 2 (DOCX 49 kb)

## Data Availability

Data used in this research are available upon request from the corresponding author.
