# Supplementary material for: Freshwater alga Raphidocelis subcapitata undergoes metabolomic changes in response to electrostatic adhesion by micrometer-sized nylon 6 particles
Source: Environ Sci Pollut Res Int. 2021 Jul 8;28(47):66901–13. doi: 10.1007/s11356-021-15300-8 (PMC8642260; doi:10.1007/s11356-021-15300-8)
Supplement: Supplementary file 1 — (PPTX 46 kb) [file 11356_2021_15300_MOESM1_ESM.pptx]

## Slide 1
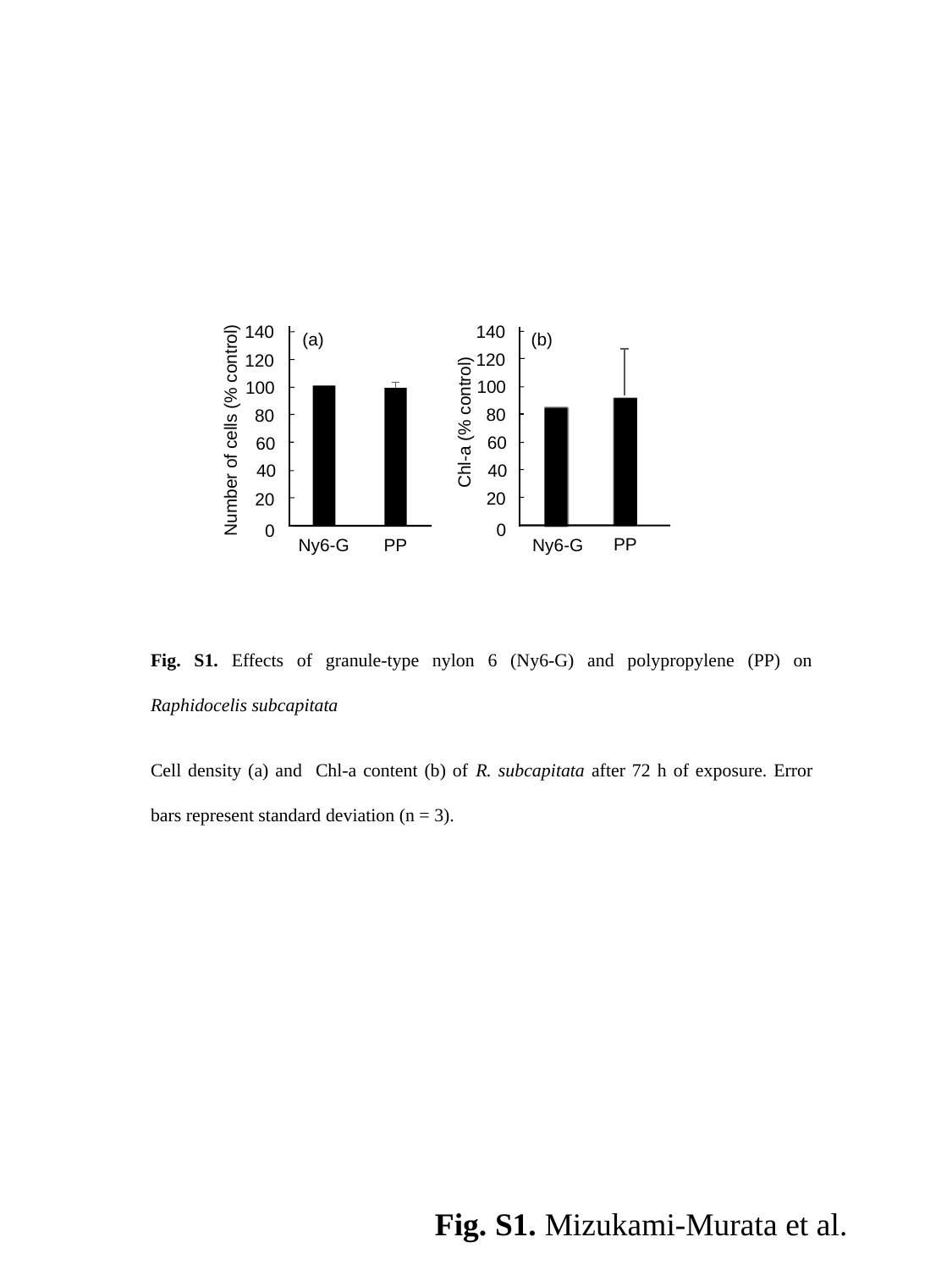

140
120
100
80
60
40
20
0
Number of cells (% control)
PP
Ny6-G
140
(a)
(b)
120
100
80
Chl-a (% control)
60
40
20
0
PP
Ny6-G
Fig. S1. Effects of granule-type nylon 6 (Ny6-G) and polypropylene (PP) on Raphidocelis subcapitata
Cell density (a) and Chl-a content (b) of R. subcapitata after 72 h of exposure. Error bars represent standard deviation (n = 3).
Fig. S1. Mizukami-Murata et al.

## Slide 2
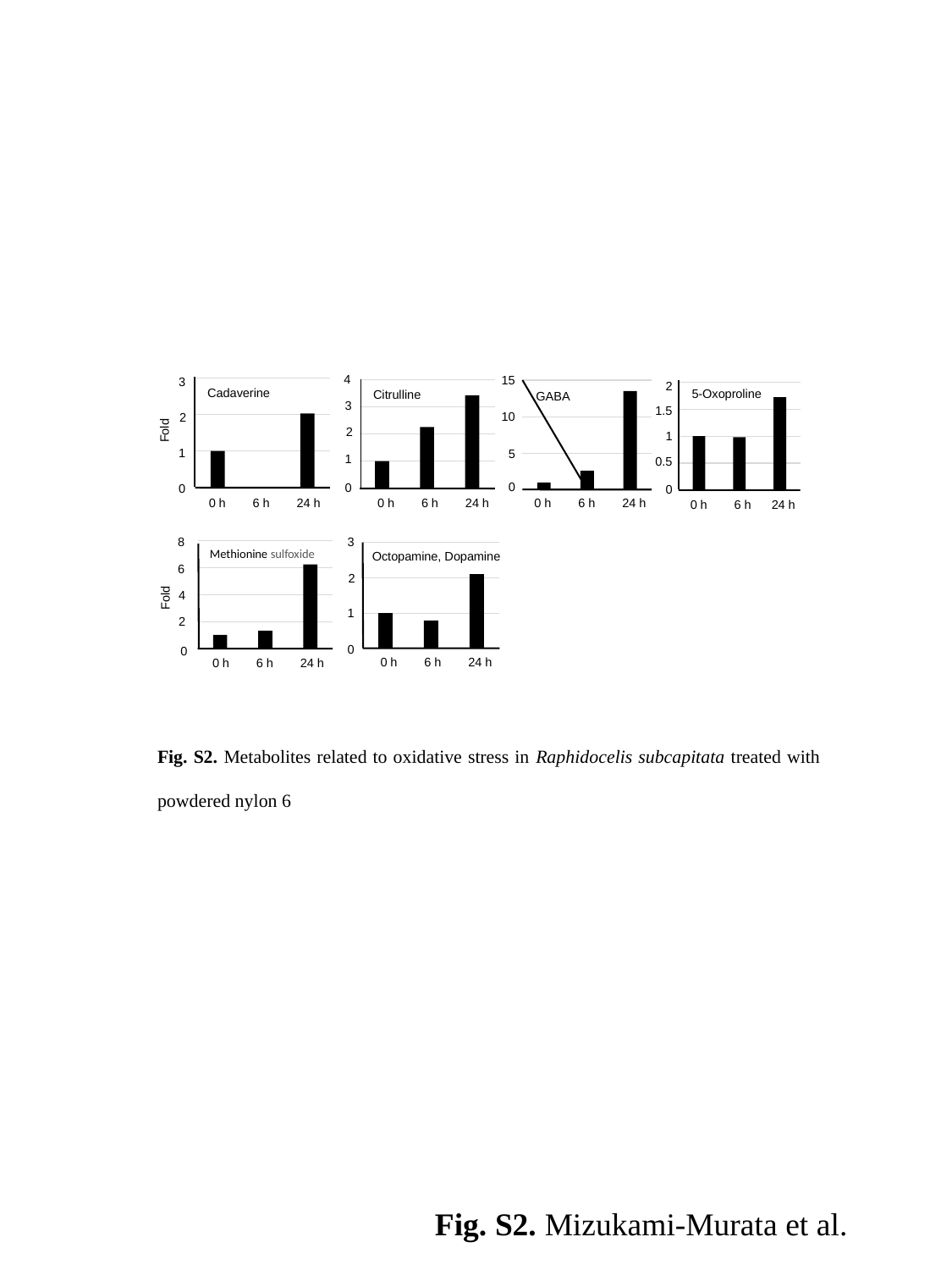

4
3
2
1
0
Citrulline
0 h 6 h 24 h
15
3
2
1
0
2
Cadaverine
5-Oxoproline
GABA
1.5
10
Fold
1
5
0.5
0
0
0 h 6 h 24 h
0 h 6 h 24 h
0 h 6 h 24 h
8
Methionine sulfoxide
6
4
2
0
0 h 6 h 24 h
3
2
1
0
0 h 6 h 24 h
Octopamine, Dopamine
Fold
Fig. S2. Metabolites related to oxidative stress in Raphidocelis subcapitata treated with powdered nylon 6
Fig. S2. Mizukami-Murata et al.
