# Supplementary material for: Freshwater alga Raphidocelis subcapitata undergoes metabolomic changes in response to electrostatic adhesion by micrometer-sized nylon 6 particles
Source: Environ Sci Pollut Res Int. 2021 Jul 8;28(47):66901–13. doi: 10.1007/s11356-021-15300-8 (PMC8642260; doi:10.1007/s11356-021-15300-8)
Supplement: Supplementary file 2 — (DOCX 49 kb) [file 11356_2021_15300_MOESM2_ESM.docx]

|  | Table S1 Metabolisms detected in *Raphidocelis subcapitata* treated with powdered Ny6 (Ny6-P) | | | | | | | | | | |
| --- | --- | --- | --- | --- | --- | --- | --- | --- | --- | --- | --- |
| No. | HMT DB | Detection methods | *m/z* | MT/RT | Relative Area | | | | | Comparative Analysis | |
|  | Compound name |  |  |  | Control | | | Treatment | Treatment | Ratio | |
|  |  |  |  |  | 0 hour | 6 hour | 24 hour | 6 hour | 24 hour | Ny6-6h  vs C-6 h | Ny6-24h  vs C-24 h |
|  |  |  |  |  | C-0h | C-6h | C-24h | Ny6-6h | Ny6-24h |  |  |
| 1 | 1-Methyl-4-imidazoleacetic acid | C | 141.067 | 8.07 | N.D. | N.D. | 1.3E-04 | N.D. | N.D. | N.A. | <1 |
| 2 | 11-Aminoundecanoic acid | C | 202.181 | 9.46 | 9.8E-03 | 1.1E-02 | 5.9E-03 | 8.5E-03 | 9.2E-03 | 0.8 | 1.6 |
| 3 | 2-Amino-2-(hydroxymethyl)-1,3-propanediol | C | 123.085 | 8.05 | 1.8E-01 | 1.0E-02 | 1.0E-02 | 3.3E-02 | 2.0E-02 | 3.3 | 1.9 |
| 4 | 2-Hydroxy-4-methylvaleric acid | A | 131.071 | 8.73 | 1.4E-03 | 1.6E-03 | 1.2E-03 | 1.2E-03 | 1.5E-03 | 0.7 | 1.2 |
| 5 | 2-Hydroxyglutaric acid | A | 147.029 | 16.60 | N.D. | N.D. | N.D. | N.D. | 1.1E-03 | N.A. | 1< |
| 6 | 2-Isopropylmalic acid | A | 175.061 | 13.53 | N.D. | 1.5E-03 | N.D. | 1.1E-03 | 3.8E-03 | 0.7 | 1< |
| 7 | 2-Oxoglutaric acid | A | 145.015 | 20.98 | N.D. | 4.8E-03 | 5.0E-03 | N.D. | N.D. | <1 | <1 |
| 8 | 2-Phosphoglyceric acid | A | 184.986 | 18.92 | 2.0E-03 | 1.9E-03 | 2.8E-03 | 1.7E-03 | 2.3E-03 | 0.9 | 0.8 |
| 9 | 3-Hydroxy-2-methyl-4-pyrone | C | 127.039 | 22.70 | 1.7E-02 | 1.3E-02 | 2.0E-02 | 2.2E-02 | 2.1E-02 | 1.7 | 1.1 |
| 10 | 3-Hydroxybutyric acid | A | 103.040 | 9.36 | 2.1E-03 | 2.5E-03 | 1.7E-03 | 2.3E-03 | 2.6E-03 | 0.9 | 1.6 |
| 11 | 3-Phenylpropionic acid | A | 149.061 | 8.65 | 3.9E-02 | 3.0E-02 | 2.7E-02 | 3.4E-02 | 3.7E-02 | 1.1 | 1.4 |
| 12 | 3-Phosphoglyceric acid | A | 184.986 | 19.40 | 3.0E-02 | 2.5E-02 | 2.6E-02 | 1.8E-02 | 3.0E-02 | 0.7 | 1.2 |
| 13 | 4-Oxopyrrolidine-2-carboxylic acid | C | 130.050 | 10.74 | 1.4E-03 | 1.2E-03 | 1.7E-03 | 1.4E-03 | 1.2E-03 | 1.2 | 0.7 |
| 14 | 4-Oxovaleric acid | A | 115.040 | 9.55 | 2.8E-03 | 2.4E-03 | 1.9E-03 | 2.4E-03 | 2.7E-03 | 1.0 | 1.4 |
| 15 | 5-Amino-4-oxovaleric acid | C | 132.066 | 7.78 | N.D. | 1.0E-03 | 2.6E-04 | 4.0E-04 | N.D. | 0.4 | <1 |
| 16 | 5-Aminoindole | C | 133.076 | 7.37 | 4.1E-04 | 4.4E-04 | 2.0E-04 | 2.9E-04 | 2.5E-04 | 0.7 | 1.2 |
| 17 | 5-Oxohexanoic acid | A | 129.056 | 8.94 | 1.3E-02 | 1.2E-02 | 1.2E-02 | 1.2E-02 | 1.5E-02 | 1.0 | 1.3 |
| 18 | 5-Oxoproline | A | 128.036 | 9.32 | 1.9E-03 | 1.6E-03 | 2.3E-03 | 1.6E-03 | 3.9E-03 | 1.0 | 1.7 |
| 19 | 6-Aminohexanoic acid | C | 132.102 | 8.14 | N.D. | N.D. | N.D. | 2.5E-01 | 1.1E-01 | 1< | 1< |
| 20 | 7-Methylxanthine | A | 165.041 | 8.51 | 5.3E-03 | 4.5E-03 | 3.9E-03 | 4.7E-03 | 5.3E-03 | 1.0 | 1.4 |
| 21 | 8-Hydroxyoctanoic acid 2-Hydroxyoctanoic acid | A | 159.103 | 7.93 | 1.5E-03 | 1.7E-03 | 8.4E-04 | 1.3E-03 | 7.6E-04 | 0.8 | 0.9 |
| 22 | Adenosine | C | 268.105 | 9.65 | N.D. | 3.9E-04 | 1.7E-04 | 3.1E-04 | 5.0E-04 | 0.8 | 3.0 |
| 23 | Adipic acid | A | 145.051 | 14.63 | N.D. | 3.6E-03 | 2.5E-03 | 3.5E-03 | 2.9E-03 | 1.0 | 1.1 |
| 24 | ADP | A | 426.023 | 10.68 | 3.7E-03 | 4.4E-03 | 8.8E-03 | 5.1E-03 | 1.0E-02 | 1.2 | 1.2 |
| 25 | Ala | C | 90.055 | 8.81 | 1.7E-02 | 1.6E-02 | 1.6E-02 | 4.7E-02 | 6.0E-02 | 2.9 | 3.8 |
| 26 | Aminoacetone | C | 74.060 | 6.66 | N.D. | N.D. | 1.9E-03 | N.D. | N.D. | N.A. | <1 |
| 27 | AMP | A | 346.058 | 9.12 | 2.3E-03 | 2.6E-03 | 2.5E-03 | 2.3E-03 | 8.2E-03 | 0.9 | 3.3 |
| 28 | Arg | C | 175.119 | 6.90 | 3.5E-02 | 1.8E-02 | 4.5E-02 | 8.2E-02 | 1.1E-01 | 4.7 | 2.5 |
| 29 | Arg-Glu | C | 304.163 | 7.28 | 3.1E-04 | N.D. | 1.1E-04 | 4.3E-04 | 7.7E-04 | 1< | 7.0 |
| 30 | Argininosuccinic acid | C | 291.130 | 9.19 | 3.4E-04 | N.D. | 1.1E-04 | 1.0E-03 | 1.4E-03 | 1< | 13 |
| 31 | Ascorbic acid | A | 175.026 | 8.35 | N.D. | 2.2E-02 | 2.6E-03 | 3.2E-02 | 1.7E-03 | 1.4 | 0.6 |
| 32 | Asn | C | 133.061 | 10.26 | 4.1E-03 | 3.8E-03 | 3.1E-03 | 5.3E-03 | 8.8E-03 | 1.4 | 2.8 |
| 33 | Asp | C | 134.045 | 11.36 | 1.9E-02 | 2.1E-02 | 3.5E-02 | 2.1E-02 | 2.4E-02 | 1.0 | 0.7 |
| 34 | ATP | A | 505.989 | 11.58 | N.D. | 3.5E-03 | 1.0E-02 | 1.1E-03 | 5.9E-03 | 0.3 | 0.6 |
| 35 | Azelaic acid | A | 187.099 | 11.88 | 1.1E-02 | 1.5E-02 | 6.9E-03 | 1.1E-02 | 5.4E-03 | 0.7 | 0.8 |
| 36 | Benzoic acid | A | 121.030 | 9.66 | 1.3E-02 | 9.4E-03 | 7.9E-03 | 1.3E-02 | 1.1E-02 | 1.4 | 1.4 |
| 37 | Biotin | A | 243.083 | 7.60 | 1.1E-03 | N.D. | 5.3E-04 | N.D. | N.D. | N.A. | <1 |
| 38 | Cadaverine | C | 103.123 | 4.80 | 2.0E-04 | 1.3E-04 | 7.3E-05 | N.D. | 1.5E-04 | <1 | 2.0 |
| 39 | CDP | A | 402.013 | 11.27 | N.D. | N.D. | 4.2E-04 | N.D. | 1.1E-03 | N.A. | 2.7 |
| 40 | Choline | C | 104.107 | 6.64 | 1.2E-03 | 1.8E-03 | 2.4E-03 | 1.5E-03 | 1.5E-03 | 0.9 | 0.6 |
| 41 | *cis*-Aconitic acid | A | 173.007 | 27.58 | N.D. | N.D. | 3.5E-03 | N.D. | N.D. | N.A. | <1 |
| 42 | Citric acid | A | 191.020 | 25.95 | N.D. | 4.4E-03 | 2.7E-02 | 3.2E-03 | 9.7E-03 | 0.7 | 0.4 |
| 43 | Citrulline | C | 176.104 | 10.86 | 1.4E-03 | 1.0E-03 | 7.8E-04 | 2.3E-03 | 2.7E-03 | 2.3 | 3.4 |
| 44 | CMP | A | 322.047 | 9.44 | N.D. | N.D. | N.D. | N.D. | 1.8E-03 | N.A. | 1< |
| 45 | CMP-*N*-acetylneuraminate | A | 613.145 | 8.22 | 8.7E-04 | 1.7E-03 | 3.2E-03 | 1.8E-03 | 2.1E-03 | 1.0 | 0.7 |
| 46 | Crotonic acid | A | 85.030 | 10.57 | N.D. | N.D. | N.D. | 1.7E-03 | N.D. | 1< | N.A. |
| 47 | Cumic acid | A | 163.076 | 8.19 | 6.1E-04 | 8.4E-04 | 7.7E-04 | 7.8E-04 | 6.3E-04 | 0.9 | 0.8 |
| 48 | Cysteine glutathione disulfide | C | 427.097 | 11.45 | 2.9E-04 | N.D. | N.D. | N.D. | 4.5E-04 | N.A. | 1< |
| 49 | Decanoic acid | A | 171.139 | 7.83 | 5.4E-03 | N.D. | 1.3E-03 | 4.7E-03 | 2.7E-03 | 1< | 2.1 |
| 50 | Diethanolamine | C | 106.087 | 7.42 | 2.5E-02 | 2.6E-02 | 1.9E-02 | 1.9E-02 | 1.5E-02 | 0.7 | 0.8 |
| 51 | Dihydroxyacetone phosphate | A | 168.991 | 12.43 | N.D. | 2.0E-03 | 6.7E-04 | 1.2E-03 | 1.2E-03 | 0.6 | 1.7 |
| 52 | Dimethylaminoethanol | C | 90.090 | 6.72 | N.D. | 3.5E-04 | N.D. | N.D. | N.D. | <1 | N.A. |
| 53 | Diphenylcarbazide | C | 243.124 | 22.86 | 2.8E-03 | 2.3E-03 | 1.8E-03 | 2.0E-03 | 2.3E-03 | 0.9 | 1.3 |
| 54 | Ethanolamine | C | 62.061 | 6.14 | 1.2E-03 | 2.4E-03 | 9.0E-04 | N.D. | 4.9E-03 | <1 | 5.5 |
| 55 | Ethanolamine phosphate | A | 140.013 | 7.92 | N.D. | N.D. | N.D. | 4.8E-03 | 1.5E-02 | 1< | 1< |
| 56 | Fructose 1,6-diphosphate | A | 338.989 | 14.37 | N.D. | 4.6E-03 | N.D. | 2.1E-03 | 3.1E-03 | 0.5 | 1< |
| 57 | Fructose 6-phosphate | A | 259.024 | 9.79 | 1.2E-03 | 3.2E-03 | 2.5E-03 | 3.7E-03 | 2.8E-03 | 1.1 | 1.1 |
| 58 | Fumaric acid | A | 115.004 | 24.94 | 3.9E-03 | N.D. | 3.0E-03 | N.D. | N.D. | N.A. | <1 |
| 59 | GABA | C | 104.071 | 7.47 | N.D. | 8.1E-03 | 7.5E-04 | 2.1E-02 | 1.0E-02 | 2.6 | 14 |
| 60 | GDP | A | 442.020 | 10.44 | N.D. | N.D. | 8.2E-04 | N.D. | N.D. | N.A. | <1 |
| 61 | GDP-mannose GDP-galactose GDP-glucose | A | 604.070 | 8.19 | N.D. | N.D. | 6.5E-04 | N.D. | 1.0E-03 | N.A. | 1.5 |
| 62 | Gln | C | 147.077 | 10.55 | 1.3E-02 | 2.4E-02 | 2.0E-02 | 3.0E-02 | 1.8E-02 | 1.2 | 0.9 |
| 63 | Glu | C | 148.061 | 10.74 | 1.9E-01 | 1.7E-01 | 2.6E-01 | 1.9E-01 | 1.5E-01 | 1.1 | 0.6 |
| 64 | Gluconolactone | C | 179.053 | 23.54 | N.D. | N.D. | N.D. | 1.3E-03 | 1.2E-03 | 1< | 1< |
| 65 | Glucose 1-phosphate | A | 259.023 | 9.96 | N.D. | 6.9E-04 | N.D. | 1.3E-03 | 1.1E-03 | 1.9 | 1< |
| 66 | Glucose 6-phosphate | A | 259.024 | 9.68 | 3.3E-03 | 1.1E-02 | 7.0E-03 | 2.3E-02 | 9.4E-03 | 2.0 | 1.4 |
| 67 | Glutaric acid Methylsuccinic acid | A | 131.036 | 16.61 | 6.4E-03 | 5.4E-03 | 4.0E-03 | 6.8E-03 | 6.9E-03 | 1.2 | 1.7 |
| 68 | Glutathione (GSSG)_divalent | C | 307.084 | 12.06 | 6.4E-03 | 1.4E-02 | 2.7E-02 | 1.7E-02 | 2.2E-02 | 1.2 | 0.8 |
| 69 | Gly | C | 76.039 | 8.08 | 2.6E-03 | 4.2E-03 | 3.7E-03 | 5.8E-03 | 1.2E-02 | 1.4 | 3.1 |
| 70 | Gly-Asp | C | 191.068 | 9.66 | 2.2E-04 | N.D. | 4.7E-04 | 4.1E-04 | 3.7E-04 | 1< | 0.8 |
| 71 | Gly-Leu | C | 189.121 | 9.57 | N.D. | N.D. | N.D. | N.D. | 2.6E-04 | N.A. | 1< |
| 72 | Glyceric acid | A | 105.020 | 10.18 | 1.7E-03 | 1.8E-03 | 1.2E-03 | 1.6E-03 | 1.8E-03 | 0.9 | 1.4 |
| 73 | Glycerol | C | 93.055 | 22.64 | 2.5E+00 | 2.8E+00 | 1.4E+00 | 1.6E+00 | 1.9E+00 | 0.6 | 1.3 |
| 74 | Glycerol 2-phosphate | A | 171.006 | 12.49 | 1.5E-01 | 2.5E-03 | 4.4E-03 | 2.6E-02 | 1.3E-02 | 10 | 2.9 |
| 75 | Glycerol 3-phosphate | A | 171.007 | 11.92 | 1.0E-02 | N.D. | 8.8E-04 | 3.8E-03 | 3.0E-03 | 1< | 3.4 |
| 76 | Glycolic acid | A | 75.009 | 12.41 | 1.7E-02 | 1.4E-02 | 8.6E-03 | 1.3E-02 | N.D. | 0.9 | <1 |
| 77 | GMP | A | 362.053 | 9.03 | N.D. | N.D. | N.D. | N.D. | 1.1E-03 | N.A. | 1< |
| 78 | GTP | A | 521.984 | 11.28 | N.D. | N.D. | 6.7E-04 | N.D. | N.D. | N.A. | <1 |
| 79 | Guanine | C | 152.056 | 8.07 | N.D. | N.D. | 4.8E-04 | 1.9E-04 | 1.0E-03 | 1< | 2.1 |
| 80 | Guanosine | C | 284.101 | 12.35 | N.D. | N.D. | 2.1E-04 | N.D. | 7.1E-04 | N.A. | 3.4 |
| 81 | Heptanoic acid | A | 129.093 | 8.46 | 1.1E-03 | N.D. | 7.6E-04 | 1.9E-03 | 7.6E-04 | 1< | 1.0 |
| 82 | Hexanoic acid | A | 115.077 | 8.77 | 2.5E-03 | N.D. | 1.1E-03 | 3.8E-03 | 1.2E-03 | 1< | 1.2 |
| 83 | Hexylamine | C | 102.129 | 7.79 | N.D. | 2.7E-04 | 1.5E-04 | N.D. | N.D. | <1 | <1 |
| 84 | His | C | 156.077 | 7.08 | 9.5E-04 | 8.1E-04 | 1.1E-03 | 3.9E-03 | 9.3E-03 | 4.8 | 8.2 |
| 85 | Homoserine | C | 120.066 | 9.86 | 1.4E-03 | 2.2E-03 | 1.2E-03 | N.D. | N.D. | <1 | <1 |
| 86 | Ile | C | 132.102 | 10.00 | 2.2E-03 | 2.2E-03 | 4.1E-03 | 9.0E-03 | 2.1E-02 | 4.0 | 5.1 |
| 87 | Isethionic acid | A | 124.992 | 11.40 | 2.2E-03 | 3.1E-03 | 1.2E-03 | 2.6E-03 | 2.9E-03 | 0.8 | 2.4 |
| 88 | Isobutyric acid Butyric acid | A | 87.046 | 9.68 | N.D. | N.D. | 6.3E-04 | 1.5E-03 | N.D. | 1< | <1 |
| 89 | Isovaleric acid DL-2-Methylbutyric acid Valeric acid | A | 101.061 | 9.08 | 1.9E-03 | N.D. | 1.3E-03 | 2.4E-03 | N.D. | 1< | <1 |
| 90 | Isovalerylalanine *N*-Acetylleucine | A | 172.099 | 7.93 | 3.7E-03 | 3.5E-03 | 1.4E-03 | 3.1E-03 | 3.4E-03 | 0.9 | 2.4 |
| 91 | Lactic acid | A | 89.025 | 10.53 | 7.0E-02 | 7.7E-02 | 6.8E-02 | 6.6E-02 | 7.9E-02 | 0.9 | 1.2 |
| 92 | Lauric acid | A | 199.171 | 7.55 | 1.3E-02 | N.D. | N.D. | 2.3E-03 | 1.1E-03 | 1< | 1< |
| 93 | Leu | C | 132.102 | 10.11 | 2.2E-03 | 2.4E-03 | 4.7E-03 | 1.3E-02 | 2.4E-02 | 5.5 | 5.2 |
| 94 | Lys | C | 147.113 | 6.66 | 2.6E-02 | 7.4E-02 | 8.3E-02 | 7.1E-02 | 1.1E-01 | 1.0 | 1.3 |
| 95 | Malic acid | A | 133.014 | 20.99 | 6.4E-02 | 7.5E-02 | 1.6E-01 | 4.1E-02 | 4.0E-02 | 0.5 | 0.2 |
| 96 | Melamine | C | 127.073 | 7.39 | 3.4E-03 | 3.2E-03 | 1.8E-03 | 3.3E-03 | 4.3E-04 | 1.1 | 0.2 |
| 97 | Met | C | 150.058 | 10.52 | 7.3E-04 | 3.2E-03 | 3.5E-03 | 8.6E-03 | 2.1E-02 | 2.7 | 6.1 |
| 98 | Methionine sulfoxide | C | 166.054 | 11.61 | N.D. | 4.5E-04 | 4.4E-04 | 6.0E-04 | 2.7E-03 | 1.3 | 6.2 |
| 99 | Mevalolactone | C | 131.070 | 22.85 | 5.8E-03 | 6.1E-03 | 4.6E-03 | 5.8E-03 | 7.0E-03 | 0.9 | 1.5 |
| 100 | Morpholine | C | 88.076 | 6.41 | N.D. | 7.9E-04 | 3.6E-04 | N.D. | 3.1E-04 | <1 | 0.8 |
| 101 | *myo*-Inositol 1-phosphate *myo*-Inositol 3-phosphate | A | 259.023 | 10.09 | N.D. | 7.4E-04 | 1.0E-03 | 1.0E-03 | N.D. | 1.4 | <1 |
| 102 | *N*,*N*-Dimethylglycine | C | 104.071 | 10.71 | N.D. | N.D. | N.D. | N.D. | 7.6E-04 | N.A. | 1< |
| 103 | *N*-(1-Deoxy-1-fructosyl)valine | C | 280.140 | 13.92 | N.D. | 4.1E-04 | N.D. | N.D. | N.D. | <1 | N.A. |
| 104 | *N*-Acetylglutamic acid | A | 188.057 | 12.94 | 1.7E-03 | 1.7E-03 | 4.8E-03 | 1.6E-03 | 1.3E-03 | 0.9 | 0.3 |
| 105 | *N*-Acetylornithine | C | 175.108 | 9.35 | 6.2E-04 | N.D. | 2.3E-04 | N.D. | 3.4E-04 | N.A. | 1.5 |
| 106 | *N*-Acetylputrescine | C | 131.117 | 8.03 | N.D. | N.D. | N.D. | 5.1E-04 | 2.6E-04 | 1< | 1< |
| 107 | *N*-Ethylmaleimide_+H_2_O | C | 144.066 | 22.64 | 1.2E-03 | 1.3E-03 | 7.0E-04 | N.D. | 1.1E-03 | <1 | 1.5 |
| 108 | *N*^6^,*N*^6^,*N*^6^-Trimethyllysine | C | 189.162 | 6.96 | 2.4E-04 | 1.9E-04 | 3.7E-04 | N.D. | 2.3E-04 | <1 | 0.6 |
| 109 | NAD^+^ | A | 662.104 | 6.52 | 9.1E-04 | 1.1E-03 | 1.3E-03 | 2.4E-03 | 3.3E-03 | 2.2 | 2.6 |
| 110 | Nicotinamide | C | 123.056 | 7.17 | 8.1E-04 | 5.2E-04 | 6.6E-04 | 4.3E-04 | N.D. | 0.8 | <1 |
| 111 | Norspermidine | C | 132.150 | 4.18 | 1.1E-03 | 1.3E-04 | 1.6E-04 | N.D. | N.D. | <1 | <1 |
| 112 | *O*-Acetylhomoserine 2-Aminoadipic acid | C | 162.077 | 10.69 | N.D. | N.D. | 1.5E-04 | N.D. | N.D. | N.A. | <1 |
| 113 | *O*-Acetylserine | C | 148.060 | 12.46 | N.D. | 7.9E-04 | 2.8E-04 | N.D. | N.D. | <1 | <1 |
| 114 | Octanoic acid | A | 143.108 | 8.22 | 4.6E-03 | N.D. | 1.4E-03 | 6.8E-03 | 2.3E-03 | 1< | 1.7 |
| 115 | Octopamine Dopamine | C | 154.087 | 8.60 | 3.0E-04 | 4.5E-04 | 3.0E-04 | 3.6E-04 | 6.3E-04 | 0.8 | 2.1 |
| 116 | Ornithine | C | 133.097 | 6.60 | 2.4E-02 | 2.5E-02 | 3.6E-02 | 4.9E-02 | 7.7E-03 | 2.0 | 0.2 |
| 117 | *p*-Toluic acid *m*-Toluic acid *o*-Toluic acid | A | 135.046 | 9.01 | N.D. | N.D. | N.D. | N.D. | 7.5E-04 | N.A. | 1< |
| 118 | Pantothenic acid | A | 218.105 | 7.58 | N.D. | N.D. | 1.9E-03 | N.D. | 7.9E-04 | N.A. | 0.4 |
| 119 | Pelargonic acid | A | 157.124 | 8.01 | 9.4E-03 | 1.9E-03 | 2.2E-03 | 9.1E-03 | 3.1E-03 | 4.8 | 1.4 |
| 120 | Phe | C | 166.087 | 10.89 | 1.3E-03 | 1.3E-03 | 2.7E-03 | 6.6E-03 | 1.8E-02 | 5.0 | 6.7 |
| 121 | Phenylpyruvic acid | A | 163.042 | 9.06 | N.D. | 1.9E-03 | N.D. | N.D. | 1.1E-03 | <1 | 1< |
| 122 | Phosphoenolpyruvic acid | A | 166.975 | 20.82 | 3.4E-03 | 3.2E-03 | 4.9E-03 | N.D. | 4.9E-03 | <1 | 1.0 |
| 123 | Phosphorylcholine | C | 184.075 | 20.97 | 2.2E-03 | 1.8E-03 | 9.0E-04 | 1.6E-03 | 5.3E-03 | 0.9 | 5.9 |
| 124 | Phthalic acid | A | 165.020 | 15.10 | 1.9E-02 | 1.9E-02 | 9.6E-03 | 1.2E-02 | 7.0E-03 | 0.7 | 0.7 |
| 125 | Piperidine | C | 86.096 | 6.63 | N.D. | N.D. | 1.6E-04 | 3.6E-04 | N.D. | 1< | <1 |
| 126 | Pro | C | 116.071 | 10.60 | 2.0E-03 | 2.2E-03 | 1.9E-03 | 8.5E-03 | 1.3E-02 | 3.8 | 6.7 |
| 127 | Putrescine | C | 89.107 | 4.53 | 3.0E-02 | 2.7E-02 | 2.5E-02 | 1.2E-02 | 2.5E-02 | 0.5 | 1.0 |
| 128 | Pyridoxamine 5'-phosphate | C | 249.064 | 10.26 | N.D. | N.D. | 1.7E-04 | 1.6E-04 | N.D. | 1< | <1 |
| 129 | Quinic acid | A | 191.056 | 7.99 | 4.6E-03 | 4.5E-03 | 4.2E-03 | 4.6E-03 | 6.5E-03 | 1.0 | 1.5 |
| 130 | Ribulose 1,5-diphosphate | A | 308.979 | 15.70 | 2.6E-03 | 3.6E-03 | 2.7E-03 | 3.8E-03 | 2.5E-02 | 1.1 | 9.3 |
| 131 | Ribulose 5-phosphate | A | 229.012 | 10.76 | N.D. | 1.1E-03 | 7.1E-04 | 1.2E-03 | 2.0E-03 | 1.1 | 2.8 |
| 132 | *S*-Adenosylmethionine | C | 399.146 | 6.89 | 1.6E-04 | 6.4E-04 | 7.6E-04 | 2.4E-04 | 6.8E-04 | 0.4 | 0.9 |
| 133 | Sebacic acid | A | 201.113 | 11.33 | N.D. | N.D. | 7.3E-04 | 1.2E-03 | N.D. | 1< | <1 |
| 134 | Sedoheptulose 7-phosphate | A | 289.033 | 9.45 | 1.3E-03 | 2.3E-03 | 2.6E-03 | 2.0E-03 | 3.0E-03 | 0.9 | 1.1 |
| 135 | Ser | C | 106.050 | 9.77 | 7.1E-03 | 5.0E-03 | 8.7E-03 | 2.0E-02 | 3.5E-02 | 4.0 | 4.0 |
| 136 | Shikimic acid | A | 173.046 | 8.07 | N.D. | 1.3E-03 | 1.1E-03 | N.D. | N.D. | <1 | <1 |
| 137 | Succinic acid | A | 117.019 | 20.62 | 2.7E-02 | 3.7E-02 | 2.7E-02 | 2.7E-02 | 4.2E-02 | 0.7 | 1.5 |
| 138 | Taurine | C | 126.022 | 22.62 | 3.5E-02 | 5.3E-02 | 1.9E-02 | 4.6E-02 | 4.5E-02 | 0.9 | 2.3 |
| 139 | Terephthalic acid | A | 165.020 | 16.33 | 2.9E-03 | 2.5E-03 | 1.4E-03 | 2.5E-03 | 2.3E-03 | 1.0 | 1.6 |
| 140 | Thiamine | C | 265.112 | 6.37 | 5.3E-02 | 4.3E-02 | 3.8E-02 | 4.0E-02 | 4.5E-02 | 0.9 | 1.2 |
| 141 | Thiamine diphosphate | A | 423.030 | 7.88 | 7.7E-04 | 1.3E-03 | 1.5E-03 | 1.3E-03 | 2.6E-03 | 0.9 | 1.8 |
| 142 | Thiamine phosphate | C | 345.081 | 10.53 | N.D. | N.D. | N.D. | N.D. | 2.3E-04 | N.A. | 1< |
| 143 | Thr | C | 120.066 | 10.30 | 5.6E-03 | 6.6E-03 | 7.8E-03 | 1.4E-02 | 2.6E-02 | 2.0 | 3.3 |
| 144 | Threonic acid | A | 135.031 | 9.07 | 3.0E-03 | 9.0E-03 | 6.0E-03 | 5.8E-03 | 3.3E-03 | 0.6 | 0.5 |
| 145 | Triethanolamine | C | 150.113 | 8.01 | N.D. | 1.7E-03 | 8.4E-04 | 1.2E-03 | 1.7E-03 | 0.7 | 2.0 |
| 146 | Trimethylamine *N*-oxide | C | 76.076 | 6.38 | N.D. | N.D. | N.D. | N.D. | 4.2E-04 | N.A. | 1< |
| 147 | Tropic acid 3-Phenyllactic acid 3-(2-Hydroxyphenyl)propionic acid *m*-Ethoxybenzoic acid *p*-Methoxyphenylacetic acid Atrolactic acid | A | 165.054 | 8.35 | 1.1E-03 | 1.5E-03 | 6.9E-04 | 1.2E-03 | 1.3E-03 | 0.8 | 1.8 |
| 148 | Trp | C | 205.098 | 10.81 | 1.2E-03 | 2.5E-03 | 3.2E-03 | 1.7E-03 | 8.0E-03 | 0.7 | 2.5 |
| 149 | Tyr | C | 182.082 | 11.15 | 1.3E-03 | 1.0E-03 | 3.9E-03 | 2.0E-03 | 6.4E-03 | 2.0 | 1.6 |
| 150 | UDP | A | 402.997 | 11.48 | N.D. | N.D. | 1.1E-03 | N.D. | 4.1E-03 | N.A. | 3.6 |
| 151 | UDP-glucose UDP-galactose | A | 565.048 | 8.51 | N.D. | 1.9E-03 | 3.0E-03 | 4.7E-03 | 9.2E-03 | 2.5 | 3.1 |
| 152 | UDP-glucuronic acid | A | 579.026 | 10.83 | N.D. | N.D. | N.D. | N.D. | 7.5E-04 | N.A. | 1< |
| 153 | UDP-*N*-acetylgalactosamine UDP-*N*-acetylglucosamine | A | 606.075 | 8.34 | N.D. | N.D. | 4.5E-04 | 1.8E-03 | 4.9E-03 | 1< | 11 |
| 154 | UMP | A | 323.029 | 9.65 | N.D. | N.D. | 5.5E-04 | N.D. | 4.1E-03 | N.A. | 7.4 |
| 155 | Urea | C | 61.040 | 21.61 | 5.1E-02 | 3.8E-02 | 2.8E-02 | 4.6E-02 | 3.7E-02 | 1.2 | 1.3 |
| 156 | Urocanic acid | C | 139.052 | 8.04 | N.D. | N.D. | 1.4E-04 | N.D. | N.D. | N.A. | <1 |
| 157 | UTP | A | 482.961 | 12.40 | N.D. | N.D. | 1.4E-03 | N.D. | 4.2E-03 | N.A. | 2.9 |
| 158 | Val | C | 118.087 | 9.80 | 7.2E-03 | 6.6E-03 | 1.1E-02 | 2.1E-02 | 4.4E-02 | 3.2 | 3.9 |
| 159 | XA0012 | A | 166.017 | 9.26 | 1.0E-03 | 1.4E-03 | 8.3E-04 | 1.1E-03 | 1.2E-03 | 0.8 | 1.5 |
| 160 | XA0017 | A | 186.114 | 7.75 | 1.4E-02 | 1.8E-02 | 5.7E-03 | 1.5E-02 | 1.4E-02 | 0.9 | 2.4 |
| 161 | XA0019 | A | 191.020 | 8.18 | 7.9E-03 | 6.6E-03 | 3.8E-03 | 1.3E-02 | 5.3E-03 | 2.0 | 1.4 |
| 162 | XA0055 | A | 368.996 | 14.08 | N.D. | 1.4E-03 | N.D. | N.D. | N.D. | <1 | N.A. |
| 163 | XA0065 | A | 445.055 | 6.90 | N.D. | N.D. | N.D. | 4.9E-03 | 1.3E-02 | 1< | 1< |
| 164 | β-Ala | C | 90.056 | 7.12 | N.D. | 7.8E-04 | 8.7E-04 | 7.6E-04 | 9.6E-04 | 1.0 | 1.1 |
| 165 | β-Hydroxyisovaleric acid | A | 117.056 | 8.97 | 7.0E-03 | 6.0E-03 | 5.2E-03 | 7.5E-03 | 7.5E-03 | 1.3 | 1.4 |
| 166 | γ-Glu-Arg_divalent | C | 152.585 | 8.48 | 2.0E-03 | 9.4E-04 | 2.0E-03 | 6.1E-03 | 5.8E-03 | 6.5 | 2.9 |
| 167 | γ-Glu-Asn | C | 262.104 | 12.67 | N.D. | N.D. | N.D. | 3.8E-04 | 1.4E-03 | 1< | 1< |
| 168 | γ-Glu-Gln | C | 276.120 | 12.95 | N.D. | N.D. | N.D. | 7.8E-04 | 6.2E-04 | 1< | 1< |
| 169 | γ-Glu-His | C | 285.121 | 8.48 | N.D. | N.D. | N.D. | 2.4E-04 | 1.8E-03 | 1< | 1< |
| 170 | γ-Glu-Ile γ-Glu-Leu | C | 261.147 | 12.74 | N.D. | N.D. | N.D. | N.D. | 1.5E-03 | N.A. | 1< |
| 171 | γ-Glu-Lys_divalent | C | 138.582 | 8.34 | N.D. | N.D. | 6.4E-05 | 3.7E-04 | 4.2E-03 | 1< | 66 |
| 172 | γ-Glu-Phe | C | 295.130 | 12.99 | N.D. | N.D. | 2.4E-04 | 4.1E-04 | 3.8E-03 | 1< | 16 |
| 173 | γ-Glu-Ser | C | 235.094 | 12.56 | N.D. | N.D. | N.D. | 7.7E-04 | 1.3E-03 | 1< | 1< |
| 174 | γ-Glu-Taurine | A | 253.050 | 8.05 | 2.4E-03 | 2.2E-03 | 1.5E-03 | 2.1E-03 | 2.8E-03 | 1.0 | 1.8 |
| 175 | γ-Glu-Trp | C | 334.142 | 13.04 | N.D. | N.D. | 3.7E-04 | N.D. | 2.0E-03 | N.A. | 5.3 |
| 176 | γ-Glu-Tyr | C | 311.124 | 13.17 | N.D. | N.D. | 5.9E-04 | N.D. | 1.4E-03 | N.A. | 2.4 |
| 177 | γ-Glu-Val | C | 247.131 | 12.62 | N.D. | N.D. | N.D. | N.D. | 1.0E-03 | N.A. | 1< |
| N.D.: Not Detected | |  |  |  |  |  |  |  |  |  |  |
| N.A.: Not Available. | |  |  |  |  |  |  |  |  |  |  |
| A: Anion | |  |  |  |  |  |  |  |  |  |  |
| C: Cation | |  |  |  |  |  |  |  |  |  |  |

| Table S2 Top 10 metabolites with the highest factor loadings detected in PC1 by Principal component analysis (PCA). | | | | | | | |
| --- | --- | --- | --- | --- | --- | --- | --- |
| No. | Compound name | *m/z* | MT/RT | Factor |  |  |  |
| 1 | γ-Glu-Asn | 262.104 | 12.67 | 0.998 |  |  |  |
| 2 | γ-Glu-His | 285.121 | 8.48 | 0.994 |  |  |  |
| 3 | Ethanolamine phosphate | 140.013 | 7.92 | 0.993 |  |  |  |
| 4 | Phe | 166.087 | 10.89 | 0.992 |  |  |  |
| 5 | Gly | 76.039 | 8.08 | 0.991 |  |  |  |
| 6 | Met | 150.058 | 10.52 | 0.990 |  |  |  |
| 7 | His | 156.077 | 7.08 | 0.989 |  |  |  |
| 8 | XA0065 | 445.055 | 6.90 | 0.989 |  |  |  |
| 9 | UDP-*N*-acetylgalactosamine UDP-*N*-acetylglucosamine | 606.075 | 8.34 | 0.987 |  |  |  |
| 10 | γ-Glu-Lys_divalent | 138.582 | 8.34 | 0.987 |  |  |  |
| 10 | Ile | 132.102 | 10.00 | 0.987 |  |  |  |
